# Insecticidal Potential of *Mentha pulegium* Essential Oil and Its Main Monoterpenes in *Drosophila melanogaster*

**DOI:** 10.3390/insects17040391

**Published:** 2026-04-03

**Authors:** Valentina Silva, Evelyn Muñoz, Constanza Reyes, Nelson Caro, Iván Montenegro, Alejandro Madrid

**Affiliations:** 1 Laboratorio de Productos Naturales y Síntesis Orgánica (LPNSO), Facultad de Ciencias Naturales y Exactas, Universidad de Playa Ancha, Leopoldo Carvallo 270, Valparaíso 2340000, Chile; silvapedrerosv@gmail.com (V.S.); reyesveraconstanza@gmail.com (C.R.); 2 Departamento de Química Orgánica, Facultad de Ciencias Químicas, Universidad de Concepción, Concepción 4070371, Chile; emunoznu@udec.cl; 3 Centro de Investigación Austral Biotech, Facultad de Ciencias, Universidad Santo Tomás, Avda. Ejercito 146, Santiago 8320000, Chile; nelsoncarofu@santotomas.cl; 4 Center of Interdisciplinary Biomedical and Engineering Research for Health (MEDING), Escuela de Obstetricia y Puericultura, Facultad de Medicina, Universidad de Valparaíso, Angamos 655, Reñaca, Viña del Mar 2520000, Chile; 5 Millennium Nucleus Bioproducts, Genomics and Environmental Microbiology (BioGEM), Avenida España 1680, Valparaíso 2390123, Chile

**Keywords:** *Mentha pulegium*, pennyroyal, mint, pulegone, insecticidal, acetylcholinesterase

## Abstract

The control of agricultural insect pests is currently based on synthetic insecticides that raise concerns about health and environmental safety. Given the need for sustainable alternatives, this study investigated the insecticidal potential of *Mentha pulegium* essential oil and its main components. The oil was obtained by hydrodistillation and characterized by GC-MS. The larvicidal and fumigant toxicity on *Drosophila melanogaster* was evaluated, in addition to exploring its in vitro and in silico inhibitory effect on the acetylcholinesterase enzyme. Chemical analysis revealed pulegone as the major compound (63.76%), followed by menthone (5.79%). In larvae, pulegone exhibited the highest activity (LC_50_ = 119.15 µg/mL), showing no significant difference compared to the positive control carvacrol. The essential oil and menthone were significantly less toxic to the larvae. In the fumigant test, the essential oil stood out for its high efficacy, achieving 100% mortality at all concentrations tested. Pulegone led the inhibition of AChE (IC_50_ = 45.88 µg/mL), supported by molecular docking simulations that showed high affinity and hydrogen bond formation with key residues of the enzyme’s active site. The study validates the use of *M. pulegium* as a promising natural alternative for the control of dipteran pests.

## Simple Summary

The use of synthetic insecticides for pest control poses environmental and health challenges, driving the search for alternatives based on natural products. In this study, we investigated the potential of pennyroyal essential oil (*Mentha pulegium*) and its main components (pulegone and menthone) to control the fruit fly *Drosophila melanogaster*. Its efficacy was evaluated in both larvae and adults through fumigant toxicity tests, and we explored how these compounds interact with the enzyme acetylcholinesterase, which is key to the insect’s nervous system. Our results show that, although pulegone is the most toxic component, the essential oil has exceptional fumigant capacity. Furthermore, through molecular docking, we confirmed that these compounds can bind to and effectively inhibit the acetylcholinesterase enzyme in the fly. These findings suggest that *M. pulegium* oil is a promising candidate for the development of effective and sustainable bioinsecticides.

## 1. Introduction

Botanical essential oils (EOs) are a topic that has gained interest and continues to grow, especially when it comes to the evaluation of their insecticidal properties. Researchers have recently focused on their potential to develop products of natural origin that can be preservatives in food commodities [1] or control disease vectors [2]. In this context, the insecticidal potential of plant secondary metabolites, especially terpenoids, is well documented [3,4]. Most studies have focused on the order Coleoptera, with fumigation being the most common method of exposure in these investigations. Diptera and other routes of exposure, such as ingestion, are less well explored [3]. However, this does not affect the increasing interest in this field, mainly motivated by the growing concern over synthetic insecticides, specifically regarding their impact on human health and environmental safety.

This paradigm shift has driven the search for novel bio-insecticides that offer significant advantages, such as reduced environmental risk and lower toxicity to non-target organisms [5]. Within this framework, *Drosophila melanogaster*, the common fruit fly, stands out as a robust biological model for toxicological screening [6], as its ease of laboratory rearing allows for high-throughput testing of insecticidal effects of EOs and their isolated constituents. In addition to this role, when acting as a pest, *D. melanogaster* mainly affects damaged or decaying fruit, and even acts as a key vector of acid rot in grapevines [7]. Similarly, species such as *D. suzukii* are a major threat to agriculture due to their ability to oviposit on healthy fruit, causing economic losses estimated at about 500 million USD [8]. This context highlights the critical need for scientific evidence to validate the use of EOs and their metabolites by linking their observable lethal effects on these dipterans with their underlying modes of action [9]. To achieve this, integrated toxicological studies are essential. While biological assays provide direct evidence of toxicity and lethal concentrations, enzymatic approaches allow for the identification of their mechanisms of action, such as acetylcholinesterase (AChE) inhibition, which is vital for understanding neurotoxic effects [10]. Furthermore, the integration of in silico studies through molecular docking offers a tool for predicting and visualizing molecular-level interactions between bioactive compounds and receptors [11]. This comprehensive strategy not only validates the biological findings but also strengthens the scientific basis for the subsequent development of standardized botanical insecticide formulations.

Therefore, this study explores the insecticidal potential of *Mentha pulegium* using *D. melanogaster*. This plant species belongs to the Lamiaceae family, native to Europe, Africa, and the Middle East, and is a medicinal plant notable for its distinct fragrance. Its leaves, whether fresh or dried, have been traditionally employed as a natural insect repellent [12]. The existing literature describes the effects of *M. pulegium* on *D. melanogaster*, with an emphasis on its ability to induce mutations. However, these data are nearly 30 years old. While they explore larvicidal toxicity and genotoxicity, it is necessary to update the available evidence and provide information on its potential as a natural insecticide through neurotoxic mechanisms, something that the study did not aim to address [13].

Furthermore, while the fumigant effects of other species within the *Mentha* genus have been established [14,15], this specific activity has not yet been determined for *M. pulegium*.

Based on the gaps identified, this study aims to evaluate the insecticidal potential of *M. pulegium* EO and its primary components through a comprehensive analysis of their larvicidal and fumigant properties. Unlike previous studies, this work integrates inhibition assays and computational molecular docking to identify their interaction within the AChE system, one of the most common mechanisms of action among insecticides.

## 2. Materials and Methods

### 2.1. General

All reagents in the investigation were obtained from Sigma-Aldrich Co. (St. Louis, MO, USA) or AK Scientific (Union City, CA, USA) and used as received, these include: pulegone (90%), menthone (>90%), carvacrol (98%), acetylcholinesterase from *Electrophorus electricus* (electric eel), 5,5-dithiobis(2-nitrobenzoic acid) (DTNB) (95%) and acetylthiocholine iodide (ATChI) (98%).

### 2.2. Essential Oil

The study specimen was collected during the spring of 2025; leaves were harvested from populations of *M. pulegium* growing in Acantilados Federico Santa María, cliffs located in the Valparaíso Region, Chile (33°03′02″ S, 71°39′25″ W; 200 m.a.s.l). The plant material was identified by Forestry Engineer Patricio Novoa, and a voucher specimen (MP-0925) was deposited at the Natural Products and Organic Synthesis Laboratory at the Universidad de Playa Ancha, Valparaíso, Chile. The EO was obtained by hydrodistillation of fresh aerial parts (500 g) for 4 h using a Clevenger-type apparatus, with 3 L of distilled water, according to the protocol previously described [16]. The obtained EO was separated from the aqueous phase, dried over anhydrous sodium sulfate (Na_2_SO_4_), and filtered. The weight of the EO was determined gravimetrically using an analytical balance with a precision of ±0.0001 g, recovering a total mass of 7.4986 g. Additionally, the volume was measured using a 10 mL graduated cylinder, reaching 8.10 mL, which allowed the calculation of the density (0.926 g/mL) as the mass-to-volume ratio at room temperature. Finally, the EO was stored in amber glass vials at 4 °C in the dark until further analysis.

### 2.3. Chemical Composition

The analysis was performed following Moller et al.’s methodology [17]. The EO was diluted with dichloromethane, and 1 µL of the sample was analyzed using a GC-MS/MS system (Hewlett-Packard 6890) coupled to a Hewlett-Packard 5973 mass-selective detector (electron ionization, 70 eV; Hewlett-Packard, Palo Alto, CA, USA). The operating conditions were as follows: injector temperature 250 °C; detector temperature 280 °C; and helium as the carrier gas at a flow rate of 1.25 mL/min. The oven temperature program started at 35 °C (held for 5 min), increased to 260 °C at a rate of 5 °C/min, and concluded with a 5 min hold at 260 °C. Chromatographic separation was achieved using an HP-5 MS capillary column (Hewlett-Packard, Palo Alto, CA, USA). Compounds were identified by comparing their mass spectra with the NIST 2021 library database, using a match value > 800 as the acceptance criterion [18]. Identification was confirmed by comparing their retention indices (RI) with literature values for the same column type or with commercial standards, where available. The RI were determined under the same operating conditions in relation to a homologous *n*-alkane series (C_8_–C_36_) by Equation (1) [17]:

RI = 100 × (n + Tr(unknown) − Tr(n)/Tr(N) − Tr(n)),(1)

where n = the number of carbon atoms in the smaller n-alkane; N = the number of carbon atoms in the larger n-alkane; and Tr = the retention time. The components’ relative concentrations were obtained by peak area normalization.

### 2.4. Rearing of Drosophila melanogaster

Adults used in this study were obtained from a stable laboratory colony maintained at the Laboratory of Synthesis and Biotransformations of Natural Products at the University of Bío-Bío, Chillán, Chile. Age-synchronized adults were allowed to mate and oviposit for 24 h on glass culture bottles containing an artificial diet. After removing the adults, first-instar larvae were collected for larvicidal assays, while young adults (3–4 days old) were used for fumigant bioassays.

The diet was prepared by mixing the following ingredients per liter of distilled water: 60 g of cornmeal (Carozzi Corp, Santiago, Chile), 16 g of agar (PhytoTech Labs, Miramar Beach, FL, USA), 44 g of sucrose (IANSA, Santiago, Chile), and 10 g of yeast (Lefersa, Santiago, Chile). The medium was supplemented with 4.2 g of potassium nitrate (KNO_3_), 4.2 g of sodium nitrate (NaNO_3_), 0.6 g of monopotassium phosphate (KH_2_PO_4_), 0.12 g of ferrous sulfate (FeSO_4_) (PhytoTech Labs, Miramar Beach, FL, USA), and 2 mL propionic acid (Sigma-Aldrich Co., St. Louis, MO, USA). The culture bottles were maintained in a VELP 215L cooled incubator (VELP Scientific, Inc., Deer Park, NY, USA) at 24 °C, with a relative humidity of ≥60% and a photoperiod of 12 h:12 h (L:D).

### 2.5. Larvicidal Assay

The larvicidal activity of *M. pulegium* EO, its major constituents, and the corresponding controls was determined following the methodology described by Muñoz-Nuñez et al. [19]. Ten first-instar larvae were placed in Petri dishes containing artificial diet (20 mL) treated with concentrations of 10, 25, 50, 100, 150, 200, and 250 μg/mL. The Petri dishes were incubated at 24 ± 1 °C with a relative humidity of ≥60%, under a photoperiod of 12:12 h (L:D). All experiments were performed in triplicate, using an untreated artificial diet as a negative control. Mortality was recorded at 72 h, and the median lethal concentration (LC_50_) was calculated using Probit regression analysis via Microsoft^®^ Excel^®^ LTSC MSO (Version 2408 Build 16.0.17932.20638).

### 2.6. Fumigant Toxicity Assay

**Figure 1:**
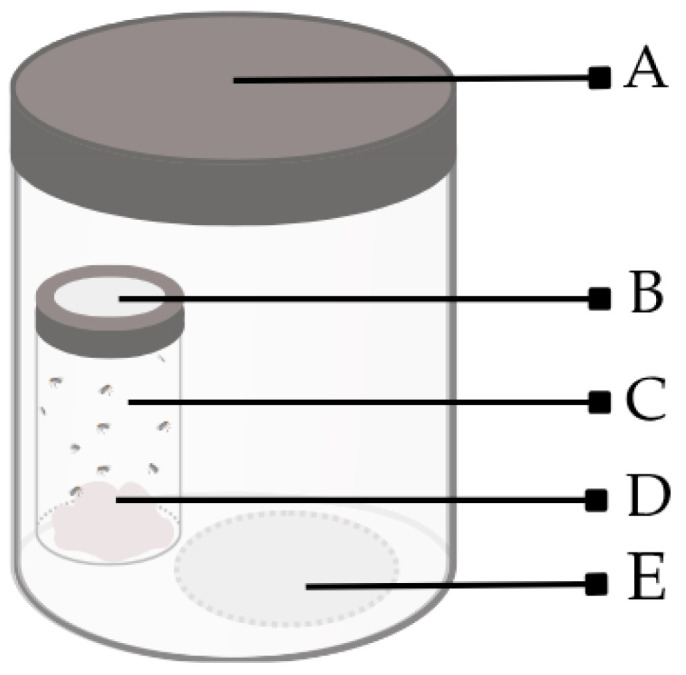
Diagram of the dual-chamber bioassay setup. **A.** A 500 mL glass chamber with a metal screw-top lid. **B.** Open-top cap covered with a fine mesh. **C.** Adult flies. **D.** Cotton soaked with 10% sugar solution **E.** Filter paper.

Fumigant activity was determined using a double-chamber system to prevent direct contact between the flies and the treatments (Figure 1) [14,20]. Samples were dissolved in acetone (20 μL) and applied to filter paper disks. After allowing the solvent to evaporate, each disk was placed at the bottom of a 500 mL glass chamber with a screw cap. Adult flies of the same age were briefly anesthetized with a CO_2_ pulse, and ten individuals were carefully selected using a fine brush and transferred to 20 mL transparent glass vials with a cotton soaked with 10% sugar solution. These vials were sealed with open-top screw caps covered with a fine mesh to allow gas exchange. Following a one-hour recovery period from anesthesia, the vials were placed inside the glass chambers containing either the treated filter paper or an acetone-treated control. Concentrations of 250, 500, 1000, 2000, and 4000 µg/L of air were applied, and all treatments were carried out in triplicate and maintained in an incubator under the conditions described above. The chambers were hermetically sealed, and mortality was recorded after 24 h. Flies were considered dead if they showed no movement after being touched with a fine brush. The median lethal concentration (LC_50_) was calculated using Probit regression analysis via Microsoft^®^ Excel^®^ LTSC MSO (Version 2408 Build 16.0.17932.20638).

### 2.7. Acetylcholinesterase Inhibition

The inhibitory activity of the samples was determined following Ellman’s method with modifications [19]. The enzyme solution (0.1 U/mL) was prepared by diluting the commercial stock in phosphate-buffered saline solution (PBS, pH 7.4). The reaction mixture comprised ATChI as the substrate and DTNB as the chromogenic reagent. The EO and compounds (pulegone, menthone, and carvacrol) were diluted in DMSO and PBS to prepare a series of twofold dilutions, starting from a maximum concentration of 250 µg/mL, followed by 125, 62.5, 31.25, and 15.625 µg/mL. In each well, 50 µL of the sample and 50 µL of the enzyme solution were pre-incubated at 37 °C for 30 min. Subsequently, 100 µL of the ATChI/DTNB mixture was added and incubated for an additional 15 min. For the control, the sample was replaced by PBS, while the blank consisted of the sample and substrate without the enzyme. Absorbance was measured at 415 nm using a Cytation 5 Multi-Mode Reader (Agilent BioTek, Santa Clara, CA, USA). Results were expressed as the median inhibitory concentration (IC_50_) calculated via sigmoidal dose–response analysis using Microsoft^®^ Excel^®^ LTSC MSO (Version 2408 Build 16.0.17932.20638). All assays were performed in triplicate.

### 2.8. Molecular Docking

The ligands evaluated in this study were retrieved from the PubChem database [21] in SMILES format and converted into three-dimensional structures using UCSF Chimera (version 1.18) [22]. During ligand preparation, polar hydrogens were added, and Gasteiger partial charges were assigned according to the Amber ff14SB force field [23,24]. The resulting geometries were subjected to energy minimization to obtain stable conformations, and the optimized structures were exported in MOL2 format for docking analysis [22]. The crystallographic structure of acetylcholinesterase from *D. melanogaster* (*Dm*-AChE; PDB ID: 6XYS; 2.46 Å resolution) was obtained from the Protein Data Bank and selected as the molecular target [25]. To validate the active site location, structural alignment was performed using the MultiSeq module in VMD (version 1.9.3), employing AChE from *Torpedo californica* (PDB ID: 1EA5) as a reference, which enabled identification of conserved catalytic residues and accurate definition of the binding region. Molecular docking simulations were conducted with AutoDock4 (version 4.2.6) [26], using the Lamarckian Genetic Algorithm for conformational sampling [27]. The search space was defined by a 24 × 24 × 24 grid box centered at X = 28.885, Y = 66.331, and Z = 14.238, encompassing the active site gorge. The receptor was treated as rigid, while ligands were allowed full torsional flexibility. For each system, 50 independent runs were performed with a maximum of 25 million energy evaluations per ligand. All simulations were carried out under conditions approximating physiological pH to reflect biologically relevant interactions.

### 2.9. Statistical

Mortality data were analyzed using the Probit model, with model adequacy confirmed via chi-square (χ^2^) tests via Microsoft^®^ Excel^®^ LTSC MSO (Version 2408 Build 16.0.17932.20638). Significant differences were determined by the non-overlap of 95% confidence intervals. Furthermore, IC_50_ values for the EO and pure constituents were compared through ANOVA and Tukey’s HSD post hoc tests (*p* < 0.05) using OriginPro 8.0 (Version 8.0724).

## 3. Results and Discussion

### 3.1. EO Composition

The EO extraction from fresh *M. pulegium* leaves produced a 1.5% (*w*/*w*) yield, resulting in a light-yellow oil. The obtained EO presented a density of 0.926 g/mL and a total volume of 8.10 mL. Its chemical composition is detailed in Table 1.

Twenty-one components were identified in the EO, representing 99.10% of its total composition. The EO was mainly characterized by pulegone (63.76%) and menthone (5.79%), which are the only components present above a 5% threshold and belong primarily to the terpenketones group. To a lesser extent, other compounds such as hexadecanoic acid (4.62%) and menthyl acetate (4.30%) were identified. Consequently, only those components exceeding the 5% threshold were selected for further testing.

This chemical composition presents differences and similarities with previous records. For instance, it differs from findings in areas near the study zone where pulegone (29.3%), menthol (28.7%), and menthone (20.4%) were reported [28]. Nonetheless, the obtained profile resembles that of *M. pulegium* collected in Greece, aligning with the findings of Pavlidou et al., where the main components were pulegone (75.7%) and menthone (10.1%) [29]. Similarly, multiple records evidence pulegone as the major compound in high concentrations, as observed in EOs of *M. pulegium* collected in Morocco (76.3%), Tunisia (61.1%), and Egypt (43.5%), among other regions [30,31].

In contrast, there are records of other populations in Iran where pulegone and menthone are marginally present (~3%), with the profile being dominated by piperitone (38%) and piperitenone (33%) [32]. The chemotypes pulegone/isomethone and piperitone/isomenthone have also been described in Sicily [33] and are linked to climate change scenarios. Increasing temperatures and reduced precipitation not only decrease primary metabolites but also alter the biosynthesis of secondary metabolites, impacting both the chemical profile and the overall yield of the *M. pulegium* EO [34]. Furthermore, high soil salinity has been associated with the upregulation of the pulegone biosynthetic pathway. Altitude also plays a critical role; *M. pulegium* plants at lower elevations exhibit a higher pulegone content, coinciding with the collection conditions, while lower temperatures and more humid conditions at higher altitudes favor the prevalence of piperitone [33].

### 3.2. Insecticidal Activity

#### 3.2.1. Larvicidal Capability

The ability of *M. pulegium* EO and its major compounds to induce mortality in first-instar larvae of *D. melanogaster* was evaluated by applying different treatments to the culture medium. The results of this assay are shown in Table 2.

Pulegone exhibited the most potent larvicidal activity (LC_50_ = 119.15 µg/mL), showing no significant difference compared to the positive control carvacrol. The latter was selected as a benchmark due to its well-documented insecticidal properties against dipterans [35,36,37]. Furthermore, as a natural constituent of *Origanum* and *Thymus* species [35,38], carvacrol provides a more chemically consistent baseline for evaluating plant-derived insecticides than synthetic alternatives. The choice of this monoterpene as a positive control is further justified by its well-established role as a multi-target toxin in insects, affecting the GABAergic system, AChE activity, carboxylesterase, glutathione-*S*-transferase, α-amylase, lipase, and trypsin, as well as cuticular hydrophobicity, among other mechanisms [39,40,41,42].

In contrast to pulegone, the *M. pulegium* EO and menthone were significantly less toxic, with LC_50_ values of 176.55 µg/mL and 185.89 µg/mL, respectively. This trend aligns with previous reports; for instance, Franzios et al. and Pavlidou demonstrated that the high toxicity of pulegone in larvae is inhibited in the presence of menthone, suggesting an antagonistic effect that modulates the overall bioactivity of the EO [13,29]. When pulegone and menthone were tested in a 7.5:1 mixture to mimic their natural relative abundance in *M. pulegium* EO, the mixture proved less effective (LC_50_ = 4.06 µL) than the EO (LC_50_ = 2.09 µL) and substantially less potent than pure pulegone (LC_50_ = 0.17 µL) against *D. melanogaster*. It is important to note that the LD_50_ values reported by these authors were obtained using a different methodology than the one applied in our study, in which the larvae were exposed directly to filter paper disks to which the treatment was applied.

#### 3.2.2. Fumigant Toxicity

Following the analysis of toxicity in larvae, the insecticidal potential of *M. pulegium* was evaluated through fumigant action assays. The results are presented in Table 3, providing a comparison of the species susceptibility when exposed to the volatile compound.

In this study, the fumigant capacity of *M. pulegium* EO surpassed the effectiveness of all pure compounds, achieving 100% mortality across all tested concentrations (LC_50_ ≤ 250 µg/L). Although larvicidal assays and the existing literature suggest an antagonistic interaction between its main components, this trend was not observed in the fumigation trials. Instead, the enhanced potency of the EO suggests that minor components, or the complex mixture as a whole, exert an additive or even synergistic effect. Consistent with the larval assays, pulegone exhibited superior fumigant performance (LC_50_ = 477.85 µg/L) compared to menthone, which did not cause mortality in adult flies (LC_50_ ≥ 4000 µg/L). These data align with Park et al., who reported an LC_50_ of 5760 μg/L for menthone against *D. suzukii* [14]. Notably, while previous studies on *D. melanogaster* reported lower LC_50_ values (0.02 and 0.19 μL/L for pulegone and menthone, respectively), those methodologies did not strictly isolate the insects from direct contact with the compounds [43]. In contrast, the double-chamber assay employed in this research ensures a strictly fumigant effect, explaining the higher toxicity thresholds observed.

The high toxicity of pulegone is attributed to its metabolic conversion into menthofuran and reactive intermediates that impair cytochrome P450 and glutathione *S*-transferase (GST) activities [44]. This mechanism is consistent with the findings of Scharf et al. [20], who reported a significant fumigant LC_50_ of 829.6 μg/L for menthofuran. In contrast to menthone, which acts primarily as a sensory repellent and exhibits toxicity only at elevated doses [45], pulegone functions as a protoxin. Consequently, its metabolic bioactivation is essential for its potent toxicological profile [46].

#### 3.2.3. In Vitro Inhibition of AChE

To further investigate the neurotoxic mechanism of action of the EO and its constituents, their inhibitory capacity against the AChE enzyme was evaluated. The in vitro enzymatic activity results, expressed as IC_50_ values, are detailed in Table 4. These data help determine whether the mortality observed in the larvicidal and fumigant assays is linked to the disruption of synaptic transmission in the insect’s nervous system [47].

The results indicate significant differences (*p* < 0.001) among the various treatments, including the positive control. Among the tested samples, the *M. pulegium* EO exhibited the lowest inhibitory activity. Nevertheless, this activity remains noteworthy, as it exceeds values reported in previous studies where an inhibition of approximately 50% was achieved at a concentration of 2 mg/mL [31]. Furthermore, these results contrast with the inhibitory capacity of *M. pulegium* ethyl acetate, *n*-butanol, and water extracts, which have been reported as inactive in similar assays; only the chloroform extract demonstrated AChE inhibition (13.16%) at a concentration of 200 µg/mL [48].

Among the compounds, pulegone showed the highest AChE inhibition, followed by menthone. In line with our larval toxicity data, pulegone emerged as the most potent inhibitor in this study. While some literature suggests that pulegone has a superior affinity for AChE and acts via irreversible inhibition [49], this remains a point of contention, as other researchers have described it as a weak inhibitor [50]. Regarding menthone, the available evidence indicates lower inhibition than carvacrol, approximately 15% versus 20% at a concentration of 0.5 mg/mL [14], which is consistent with our findings. Consequently, menthone is also categorized as a weak inhibitor by other authors [50].

### 3.3. Molecular Docking

The molecular docking results (Table 5) demonstrated favorable binding affinities of the evaluated compounds toward the active site of AChE from *D. melanogaster* (PDB: 6XYS). Among the analyzed ligands, pulegone exhibited the highest thermodynamic stability, with a binding free energy (ΔG) of −7.6 kcal/mol, whereas menthone showed a ΔG value of −6.7 kcal/mol.

**Figure 2:**
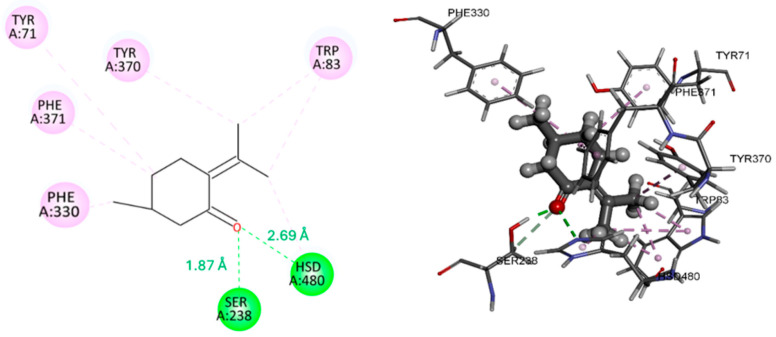
2D and 3D complex of pulegone and *Dm*-AChE.

**Figure 3:**
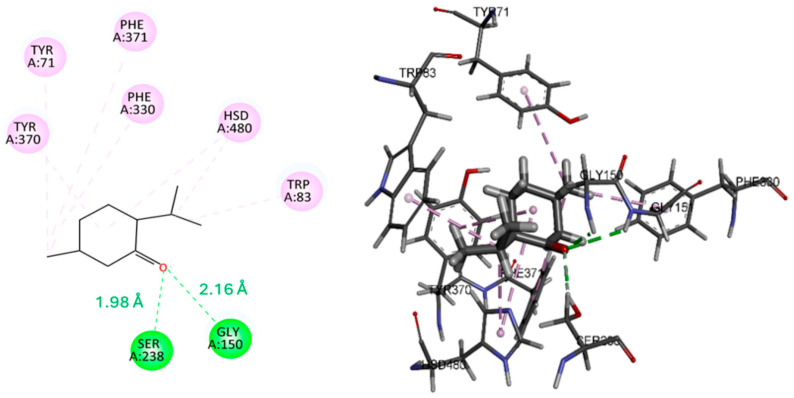
2D and 3D complexes of menthone and *Dm*-AChE.

As shown in Figure 2, pulegone formed hydrogen bond interactions with Ser238 (1.87 Å) and Hsd470 (2.69 Å), contributing to its stabilization within the catalytic gorge. In contrast, menthone established hydrogen bonds with Gly150 (2.16 Å) and Ser238 (1.98 Å) (Figure 3), the latter being a key residue of the catalytic triad responsible for acetylcholine hydrolysis [51]. The short hydrogen bond distance observed for the menthone-Ser238 interaction suggests a potential interference with the catalytic mechanism. Both ligands displayed extensive π–π and π–alkyl interactions with conserved aromatic residues, including Tyr71, Tyr370, Phe330/Phe371, and Trp83, which are located within the aromatic gorge and the peripheral anionic site. Although a recent study by Islam et al. [52] reported different interaction sites, those findings pertain to the human AChE model (PDB: 4EY7). In our study, the identified residues (Tyr71, Tyr370, Phe330, and Trp83) are specific to the *Drosophila melanogaster* enzyme (PDB: 6XYS), representing functional equivalents in the insect’s binding pocket that are critical for ligand stabilization.

Overall, the docking analysis suggests that both menthone and pulegone may act as potential inhibitors of *D. melanogaster* AChE by occupying the active site gorge and interacting with catalytically and structurally relevant residues. The stronger binding affinity observed for pulegone is consistent with previous reports indicating that monoterpenes can effectively interact with hydrophobic enzymatic cavities and inhibit acetylcholinesterase activity [14,53]. Furthermore, our binding energy results are in agreement with those reported by Amara et al. [54], who found similar binding affinities for pulegone and menthone, reinforcing the reliability of our docking model and the potential of these compounds as effective enzyme inhibitors.

## 4. Conclusions

This research demonstrates that the *M. pulegium* EO and its main component, pulegone, have high insecticidal and fumigant potential against *D. melanogaster*. The EO exhibited exceptional fumigant potency against adult flies, achieving 100% mortality at all concentrations evaluated. Likewise, of the monoterpenes evaluated individually, pulegone proved to be the most active agent in both larvicidal and fumigant assays. Furthermore, the inhibition of AChE was confirmed as a plausible mechanism of the observed toxicity, supported by computer simulations. In summary, *M. pulegium* EO is a highly effective pest control agent and a viable candidate for the development of sustainable bioinsecticides; however, its application in open fields or food storage environments must be accompanied by rigorous toxicological evaluations. Future formulations should prioritize encapsulation systems and ensure their safety for human handling.

## Figures and Tables

**Table 1:** Chemical composition of the EO obtained from leaves of *M. pulegium*.

Nº	Components	Area ^a^ (%)	RI ^b^	RL ^c^	Identification
1	*p*-Menth-3-en-8-ol	0.82	1113	1113	RL, MS
2	Menthone	5.79	1131	1131	RL, MS, Co
3	Isopulegol	1.83	1147	1147	RL, MS, Co
4	Pulegone	63.76	1216	1216	RL, MS, Co
5	Isomenthyl acetate	1.41	1280	1280	RL, MS
6	Menthyl acetate	4.30	1294	1294	RL, MS, Co
7	Piperitenone	0.39	1340	1340	RL, MS, Co
8	*α*-Caryophyllene	0.53	1414	1414	RL, MS, Co
9	Dodecanoic acid	1.05	1543	1543	RL, MS
10	Caryophyllene oxide	2.29	1572	1572	RL, MS, Co
11	Tetradecanoic acid	3.20	1743	1743	RL, MS
12	Hexadecanoic acid	4.62	1943	1943	RL, MS
13	*trans*-13-Octadecenoic acid	1.41	2164	2164	RL, MS
14	*cis*-13-Octadecenoic acid	1.14	2178	2178	RL, MS
15	Oleic Acid	1.76	2180	2180	RL, MS
16	Eicosanoic Acid	1.70	2366	2366	RL, MS
17	Oleamide	0.20	2374	2375	RL, MS
18	Cannabidiol	0.74	2384	2385	RL, MS
19	Tetracosane	0.99	2400	2400	RL, MS
20	Pentacosane	0.69	2500	2500	RL, MS
21	Hexacosane	0.48	2600	2600	RL, MS
	Total identified	99.10			
	Oxygenated monoterpenes	72.59			
	Monoterpene esters	5.71			
	Hydrocarbon sesquiterpenes	0.53			
	Oxygenated sesquiterpenes	2.29			
	Phenols	0.74			
	Hydrocarbon alkane	2.16			
	Fatty acid derivatives	0.20			
	Fatty acid	14.88			

^a^ Area: surface area of GC peak; ^b^ RI: experimental retention index for non-polar column; ^c^ RL: bibliographic retention index for non-polar column, MS: mass spectra; Co: co-elution with standard compounds available in our laboratory.

**Table 2:** Mortality of *D. melanogaster* larvae exposed to *M. pulegium* EO and its main compounds.

Treatment	LC_50_ (µg/mL)	95% CI (µg/mL)		Slope ± SE	χ^2^
		Lower	Upper		
*M. pulegium* EO	176.55 ^b^	172.53	180.66	2.22 ± 0.17	2.26
Pulegone	119.15 ^a^	111.19	127.67	1.60 ± 0.09	0.14
Menthone	185.89 ^b^	173.48	199.18	2.92 ± 0.24	4.36
Control +	119.9 ^a^	117.18	122.71	1.88 ± 0.03	1.99

CI: confidence limits; Control +: positive control carvacrol; χ^2^: chi-square test. Different letters in the superscript in the same column represent significant differences.

**Table 3:** Fumigant toxicity of *M. pulegium* EO and its primary constituents against *D. melanogaster* adults at 24 h.

Sample	LC_50_ (µg/L)	95% CI (µg/L)		Slope ± SE	χ^2^
		Lower	Upper		
*M. pulegium* EO	<250 ^a^	n.a	n.a	n.a	n.a
Pulegone	477.85 ^b^	472.76	482.99	4.20 ± 0.4	3.31
Menthone	>4000 ^d^	n.a	n.a	n.a	n.a
Control +	1290.27 ^c^	1200.38	1380.38	1.36 ± 0.27	1.21

CI: confidence limits; Control +: positive control carvacrol; χ^2^: chi-square test; n.a: not available. Different letters in the superscript in the same column represent significant differences.

**Table 4:** In vitro inhibitory effect of *M. pulegium* EO and major compounds on AChE enzyme.

Sample	IC_50_ ± SE (µg/mL)
*M. pulegium* EO	112.78 + 0.93 ^d^
Pulegone	45.88 + 0.95 ^a^
Menthone	68.57 + 0.90 ^c^
Control +	59.46 + 0.28 ^b^

Control +: positive control carvacrol. Results are expressed as mean ± standard error. Different letters in the superscript in the same column represent significant differences.

**Table 5:** Binding energies and main protein–ligand interactions of menthone and pulegone with *Dm*-AChE (PDB: 6XYS), obtained by molecular docking.

Compound	Binding Energy ΔG [Kcal/mol]	Hydrogen Bonds (Distance Å)	Interactions π and Alkyl
Pulegone	−7.6	Ser238 (1.87) Hsd470 (2.69)	Tyr71; Tyr370; Phe371; Trp83.
Menthone	−6.7	Gly150 (2.16) Ser238 (1.98)	Tyr71; Tyr372; Tyr370; Phe330; Trp83.

## Data Availability

The original contributions presented in this study are included in the article. Further inquiries can be directed to the corresponding authors.
